# Bayesian risk profiling of soil-transmitted helminth infections and estimates of preventive chemotherapy for school-aged children in Côte d'Ivoire

**DOI:** 10.1186/s13071-016-1446-0

**Published:** 2016-03-21

**Authors:** Richard B. Yapi, Frédérique Chammartin, Eveline Hürlimann, Clarisse A. Houngbedji, Prisca B. N’Dri, Kigbafori D. Silué, Jürg Utzinger, Eliézer K. N’Goran, Penelope Vounatsou, Giovanna Raso

**Affiliations:** Unité de Formation et de Recherches Biosciences, Université Félix Houphouët-Boigny, Abidjan, Côte d’Ivoire; Département Mode de Vie, Maladies Tropicales et Emergentes, Centre Suisse de Recherches Scientifiques en Côte d’Ivoire, Abidjan, Côte d’Ivoire; Department of Epidemiology and Public Health, Swiss Tropical and Public Health Institute, Basel, Switzerland; University of Basel, Basel, Switzerland; Unité de Formation et de Recherche Sciences de la Nature, Université Nangui Abrogoua, Abidjan, Côte d’Ivoire

**Keywords:** Bayesian modelling, Côte d’Ivoire, Hookworm, Risk profiling, School-aged children, Soil-transmitted helminthiasis

## Abstract

**Background:**

Soil-transmitted helminthiasis affects more than a billion people in the world and accounts for a global burden of 5.1 million disability-adjusted life years. The objectives of this study were (i) to map and predict the risk of soil-transmitted helminth infections among school-aged children in Côte d’Ivoire; (ii) to estimate school-aged children population-adjusted risk; and (iii) to estimate annual needs for preventive chemotherapy.

**Methods:**

In late 2011/early 2012, a cross-sectional survey was carried out among school-aged children in 92 localities of Côte d’Ivoire. Children provided a single stool sample that was subjected to duplicate Kato-Katz thick smears for the diagnosis of soil-transmitted helminths. A Bayesian geostatistical variable selection approach was employed to identify environmental and socioeconomic risk factors for soil-transmitted helminth infections. Bayesian kriging was used to predict soil-transmitted helminth infections on a grid of 1 × 1 km spatial resolution. The number of school-aged children infected with soil-transmitted helminths and the amount of doses needed for preventive chemotherapy according to World Health Organization guidelines were estimated.

**Results:**

Parasitological data were available from 5246 children aged 5–16 years. Helminth infections with hookworm were predominant (17.2 %). *Ascaris lumbricoides* and *Trichuris trichiura* were rarely found; overall prevalences were 1.9 % and 1.2 %, respectively. Bayesian geostatistical variable selection identified rural setting for hookworm, soil acidity and soil moisture for *A. lumbricoides*, and rainfall coefficient of variation for *T. trichiura* as main predictors of infection. The estimated school-aged children population-adjusted risk of soil-transmitted helminth infection in Côte d’Ivoire is 15.5 % (95 % confidence interval: 14.2–17.0 %). We estimate that approximately 1.3 million doses of albendazole or mebendazole are required for school-based preventive chemotherapy, and we provide school-aged children-adjusted risk aggregated at health district level.

**Conclusion:**

We provide the first soil-transmitted helminthiasis risk profile for entire Côte d’Ivoire, based on a robust Bayesian geostatistical framework. Our model-based estimates of treatment needs and risk maps on health district level may guide the national control program in spatial targeting of annual interventions.

**Electronic supplementary material:**

The online version of this article (doi:10.1186/s13071-016-1446-0) contains supplementary material, which is available to authorized users.

## Background

More than one billion people were estimated to be infected with soil-transmitted helminths (i.e. *Ascaris lumbricoides*, hookworm and *Trichuris trichiura*) in 2010 [1]. The majority of soil-transmitted helminth infections occur in Asia, sub-Saharan Africa and Latin America. Indeed, more than 90 % of the global burden due to soil-transmitted helminthiasis, which account for 5.1 million disability-adjusted life years (DALYs), are concentrated in this part of the world [2]. Infections primarily occur in remote rural and deprived urban settings among poorest communities in tropical and sub-tropical countries. Inappropriate hygiene practices, lack of access to clean water, improved sanitation, and health facilities are the main risk factors associated to the persistence of these infections [3–7]. Preventive chemotherapy with a strong focus on school-aged children is one of the main pillars of the global strategy against soil-transmitted helminthiasis [8]. However, this strategy alone does not prevent people from reinfection unless clean water, improved sanitation and adequate hygiene behaviour are available, used and practiced [9, 10].

Approaches, combining geographical information systems (GIS), remote sensing and Bayesian geostatistics, have been developed and applied in the last decade to model and predict the risk of helminth infections at high spatial resolution [11–14]. These efforts and resulting risk maps facilitate spatial targeting of control efforts in the context of resources scarcity [15]. Côte d’Ivoire is endemic to multiple helminth infections [16–19]. A particularly rich vein of investigation have been conducted in the mountainous western part of the country, including spatially explicit risk profiling [20–22]. In the Man area, elevation and land cover were identified as important predictors explaining the geographic variation of hookworm infections [11]. Yet, there is a paucity of studies investigating risk factors that explain the spatial distribution of soil-transmitted helminths at a national scale.

We present findings from a geostatistical analysis of soil-transmitted helminth infection data that were obtained from the first national school-based survey in Côte d’Ivoire. The aims of this study were (i) to map and predict the spatial distribution of soil-transmitted helminth infections in the school-aged population within a Bayesian geostatistical framework; (ii) to estimate school-aged children population-adjusted risk; and (iii) to calculate annual need for preventive chemotherapy according to guidelines put forward by the World Health Organization (WHO).

## Methods

### Ethics, consent and permissions

Ethical clearance was obtained from the ethics committees of Basel (EKBB, reference no. 30/11) and Côte d’Ivoire (reference no. 09-2011/MSHP/CNER-P). In addition, permission for the study and for the school survey was obtained from the Ministry of National Education. Heads of the health districts, education authorities, school directors and teachers of the selected schools were informed about the purpose and procedures of the study. Written informed consent was obtained from the parents/guardians of children, whereas children from consenting parents/guardians additionally assented orally. Children could withdraw from the study at any time without further obligation. Parasitological and questionnaire data were coded and treated confidentially. All study participants, regardless of soil-transmitted helminth infection status, were given a single dose of albendazole (400 mg single dose). Additionally, those children found with *Schistosoma mansoni* infection received praziquantel (40 mg/kg).

### Study design and population

Details of the study area and population surveyed have been described elsewhere [23]. In brief, the study was carried out during the dry season, from November 2011 to February 2012, and enrolled children from 93 schools in Côte d’Ivoire. We designed a cross-sectional survey using a lattice plus close pairs design [24]. From each school, at least 60 children were selected to participate, which exceeds the minimum sample size recommended by WHO, for collection of baseline information on helminth prevalence and intensity in the school-aged population within large-scale surveys [25].

### Field and laboratory procedures

The field and laboratory procedures have been detailed previously [23]. To summarise, children were invited to provide a sample of their fresh morning stool put in a 120 ml plastic container that was distributed in advance. A unique identification number was assigned to each child. Stool samples were transferred to health laboratories and processed the same day. Duplicate Kato-Katz thick smears were prepared from each stool, using standard 41.7 mg plastic templates [26, 27]. After a clearing time of 30–45 min, the thick smears were examined under a light microscope by experienced laboratory technicians. The number of helminth eggs was enumerated and recorded for each species separately. Ten percent of the Kato-Katz thick smears were randomly selected for quality control and re-examined by a senior microscopist [28]. Infection was defined as the presence of at least one helminth egg on one of the two Kato-Katz thick smears examined for each child.

### Environmental and socioeconomic predictors

Various environmental and socioeconomic proxies, known to favour helminthiasis risk, were considered as potential explanatory variables for building predictive geostatistical risk models for the three major soil-transmitted helminth infections analysed in this study. A summary description of these variables is presented in Table 1. In brief, we considered 11 environmental proxies; namely, day and night mean land surface temperatures (LST day and LST night), mean difference of LST between day and night (LST diff), land cover, normalized difference vegetation index (NDVI), yearly rainfall estimates and their coefficient of variation (rainfall CV), altitude, soil acidity (pH), soil moisture and a variable accounting for the ecological zone. Moreover, we included variables that accounted for rural/urban setting, the human influence index (HII), the percentage of households with improved sanitation and households with improved access to drinking water.

**Table 1:** Data sources and properties of environmental and socioeconomic variables explored to model the soil-transmitted helminth infection risk in Côte d’Ivoire

Data type	Source	Temporal resolution	Temporal coverage	Spatial resolution
Environmental				
Day land surface temperature (LST)	MODIS/Terra^a^	8 days	2011	1 km
Night land surface temperature (LST)	MODIS/Terra^a^	8 days	2011	1 km
LST difference	Derived from LST	8 days	2011	1 km
	(day LST - night LST)			
Land cover	MODIS/Terra^a^	Yearly	2011	1 km
Normalized difference vegetation index	MODIS/Terra^a^	16 days	2011	1 km
Rainfall	ADDS^b^	10 days	2011	8 km
Rainfall coefficient of variation (CV)	Derived from rainfall	10 days	2011	1 km
	(standard deviation/mean)			
Altitude	DEM^c^			1 km
Soil acidity (pH)	WISE3^d^			10 km
Soil moisture	WISE3^d^			10 km
Ecological zone	ISODATA^e^		2000-2008	1 km
Socioeconomic				
Urban/rural	GRUMP^f^		1995	1 km
Human influence index (HII)	LTW^g^		2005	1 km
Improved sanitation/drinking water	Bayesian kriging of DHS^h^, MICS^i^, and WHS^j^ sanitation data with urban/rural as covariate		1994-2011	1 km

^a^Moderate Resolution Imaging Spectroradiometer (MODIS). Available at: https://lpdaac.usgs.gov/ (accessed: 1 March 2015)

^b^Africa Data Dissemination Service (ADDS). Available at: http://earlywarning.usgs.gov/adds/ (accessed: 1 March 2015)

^c^ Digital elevation model (DEM). Available at: http://eros.usgs.gov/ (accessed: 1 March 2015)

^d^ISRIC-WISE database (WISE3). Available at: http://www.isric.org/ (accessed: 1 March 2015)

^e^Calculated with the Iterative Self-Organizing Data Analysis Technique [41]

^f^Global Rural–urban Mapping Project version 1 (GRUMPv1): Urban Extents Grid. Center for International Earth Science Information Network (CIESIN), International Food Policy Research Institute (IFPRI), The World Bank and Centro Internacional de Agricultura Tropical (CIAT). Available at: http://sedac.ciesin.columbia.edu/data/collection/grump-v1 (accessed: 1 March 2015)

^g^Last of the Wild Project version 2, 2005 (LWP-2): Global Human Influence Index (HII) dataset (geographic)

Wildlife Conservation Society International Earth (WCS) and Center for International Earth Science Information Network (CIESIN). Available at: http://sedac.ciesin.columbia.edu/data/set/wildareas-v2-human-influence-index-geographic (accessed: 1 March 2015)

^h^Demographic and Health Surveys. Available at: http://www.measuredhs.com (accessed: 1 March 2015)

^i^Multiple Indicator Cluster Surveys. Available at: http://www.childinfo.org/mics.html (accessed: 1 March 2015)

^j^World Health Surveys. Available at: http://www.who.int/healthinfo/survey/en/index.html (accessed: 1 March 2015)

### Statistical analysis

We modelled soil-transmitted helminth infection risks within a standard Bayesian geostatistical framework and used Markov chain Monte Carlo (MCMC) simulations algorithms to estimate model parameters [29]. In short, the prevalence of each infection at a given location is modelled on the log*it* scale as a linear function of rigorously chosen explanatory variables and a Gaussian spatial process that accounts for residual spatial correlation not otherwise captured by the covariates. The spatial structure of this latent process is introduced through a variance-covariance matrix defined as an exponential function of the distances between pairs of survey locations.

The choice of explanatory variables that define the final model was carried out with a Bayesian geostatistical variable selection approach as preliminary analysis. Such a procedure allows to explore all possible models, while accounting for spatial correlation in the data based on variable posterior probability [30]. In brief, we performed a stochastic search variable selection [31] within a geostatistical framework that explores the inclusion of a predictor in the model by multiplying each regression coefficient by a binary indicator variable that indicates the presence or absence of the predictor in the model. In order to allow a rigorous selection of predictors that are categorised, we considered “normal mixture of inverse Gamma distributions with parameter expansion” priors [32] for the regression coefficients. Continuous potential predictors were either standardised or categorised. Non-spatial univariate associations were investigated and the best functional form was chosen according to the best goodness of fit measured by the Akaike information criterion (AIC).

Prediction of the risk was carried out via Bayesian kriging [33] for more than 350,000 pixels representing a fine grid laid over Côte d’Ivoire (at 1 × 1 km spatial resolution). Our final models were validated by assessing the predictive ability, including predictive uncertainty. Each model was fitted on a random sample representing 80 % of the full dataset (72 locations) and model-based predictions of the remaining 20 % of the dataset (20 locations) were compared with observed prevalences. Thus, the mean error (ME) was calculated to assess the predictive ability, while the sum of the standard deviation (SD) of the predictive distributions measured the predictive uncertainty. The goodness of fit measure was given by the deviance information criterion (DIC) [34]. Further details on model specification and implementation of variable selection and model fit are provided in the additional information (see Additional file 1).

### Estimated needs for preventive chemotherapy

WHO recommends treatment of all school-aged children twice a year in high-risk communities (prevalence of any soil-transmitted helminth ≥ 50 %), and once every year in moderate-risk communities (prevalence of any soil-transmitted helminth infection between 20 and 50 %) [35]. To estimate the number of deworming tablets required for preventive chemotherapy on a yearly basis, we defined the soil-transmitted helminth risk level of each health district according to the school-aged children population-adjusted risk. To determine school-aged children population-adjusted risks, we first calculated the estimated soil-transmitted helminth prevalence for sample of the predictive distribution using a simple probabilistic model of combined infection divided by a factor of 1.06. In details, as the prevalences of the three species of soil-transmitted helminth are reported separately here, and given that they can occur simultaneously within a community, the number of preventive chemotherapy maybe somehow overestimated. To correct this overestimation, we applied the method described by de Silva and Hall in 2010 to accurately estimate the amount of treatments needed [36]. Then, we estimated the number of infected school-aged children with any soil-transmitted helminth infection at each prediction pixel by multiplying the predicted risk by the number of children aged 5–15 years sourced from the Afripop database (available upon request at: www.afripop.org). Finally, we obtained school-aged children adjusted risk by summing up the predicted number of infected children over the health districts, divided by the total population.

## Results

**Fig. 1:**
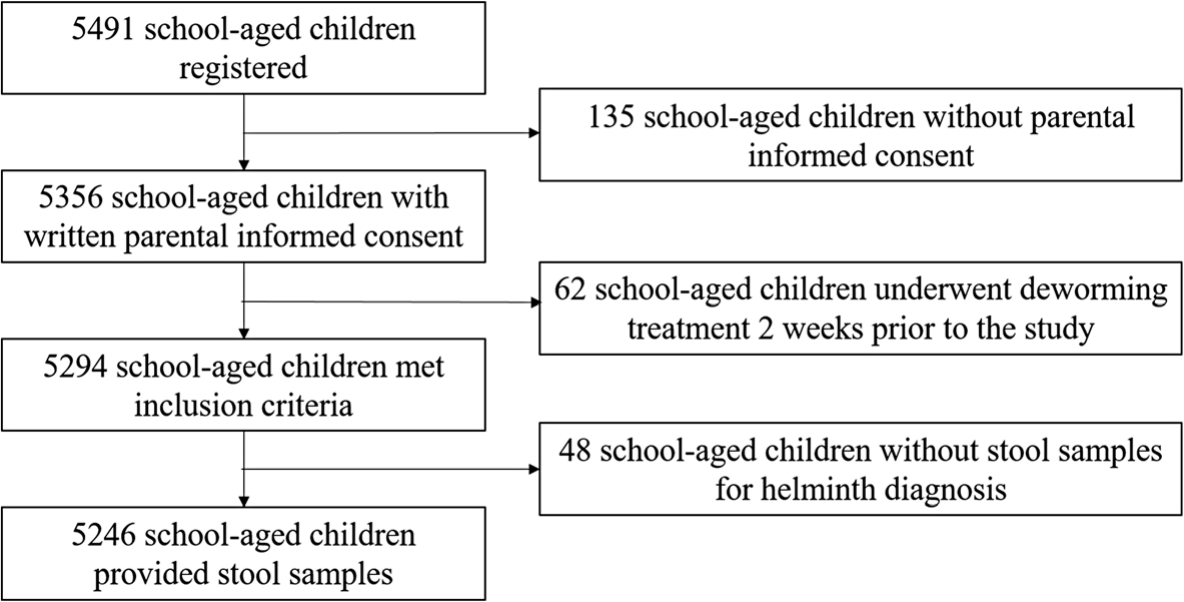
Flow chart showing the cross-sectional study compliance considered in the analysis. The study was carried out in 93 schools in Côte d’Ivoire, late 2011/early 2012

In the 93 selected schools, we invited 5491 children to provide a fresh morning stool sample for the diagnosis of soil-transmitted helminth infection. Overall, 5246 children (95.5 %) in 92 schools met our inclusion criteria. Reasons for exclusion were either absence of written parental informed consent (2.5 %) or recent deworming within two weeks prior to our survey, as reported by one school director (1.1 %) (Fig. 1). Enrolled children had an age between 5 and 16 years with a mean of 9.8 years.

**Fig. 2:**
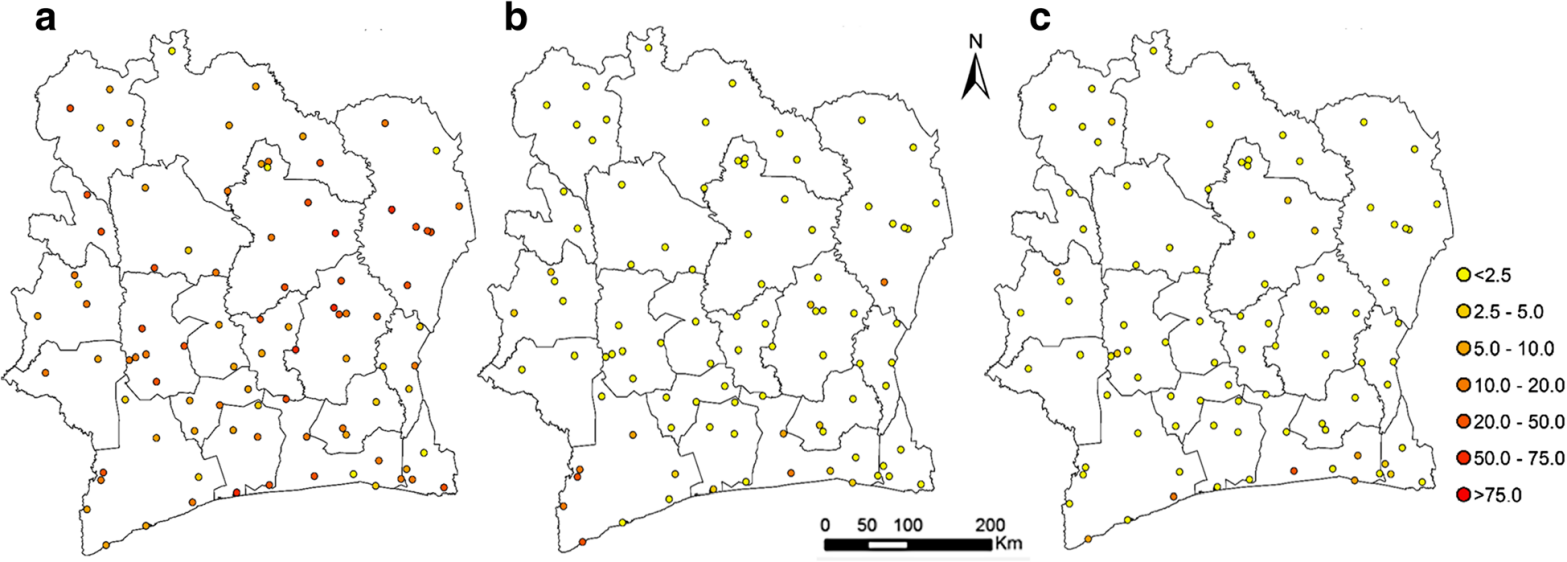
Observed soil-transmitted helminth prevalence in Côte d’Ivoire, late 2011/early 2012. **a**, hookworm; **b**, *A. lumbricoides*; **c**, *T. trichiura*

Microscopic examination of stool samples revealed eggs of soil-transmitted helminths among 1 000 children (19.1 %). Table 2 summarises soil-transmitted helminth species-specific prevalences. In short, hookworm infections were predominant with an overall prevalence of 17.2 %. *A. lumbricoides* and *T. trichiura* were rarely found; overall prevalences were 1.9 % and 1.2 %, respectively. Figure 2 shows the distribution of the observed prevalence of soil-transmitted helminth infections in Côte d’Ivoire.

**Table 2:** Soil-transmitted helminth species-specific prevalences found among school-aged children in Côte d’Ivoire, late 2011/early 2012

Soil-transmitted helminth	Children examined	Infected (%)	95 % CI^a^
Overall	5,246	1,000 (19.1)	18.0, 20.1
Hookworm	5,246	903 (17.2)	16.2, 18,2
*A. lumbricoides*	5,246	97 (1.9)	1.5, 2.2
*T. trichiura*	5,246	64 (1.2)	0.9, 1.5

^a^ *CI* confidence interval

Results of the Bayesian geostatistical variable selection are summarised in Table 3. For hookworm infections, the model with the highest posterior probability (8.9 %) selected rural/urban setting. Soil acidity and soil moisture were selected with a model posterior probability of 3.2 % to model *A. lumbricoides* infection risk. For *T. trichiura* infection, the model including rainfall CV was selected with a posterior probability of 0.4 %. Furthermore, the covariates selected also presented the highest posterior inclusion probability, in excess of 50 %. The corresponding selected covariates were chosen to build predictive risk models specific to each of the three soil-transmitted helminth species.

**Table 3:** Results of the geostatistical variable selection. Models selected with the highest model posterior probability are presented, together with posterior inclusion probability of each explored predictor

	Selected model with predictor posterior inclusion probability		
Predictor	Hookworm	*A. lumbricoides*	*T. trichiura*
Day land surface temperature (LST)^a,b,c^	Not selected (27.6 %)	Not selected (33.0 %)	Not selected (40.6 %)
Night land surface temperature (LST)^b,c^	Not selected (12.9 %)	Not selected (23.1 %)	Not selected (23.9 %)
LST difference	Not selected (10.6 %)	Not selected (22.2 %)	Not selected (24.7 %)
Land cover^a,b,c^	Not selected (18.2 %)	Not selected (16.3 %)	Not selected (31.3 %)
Normalized difference vegetation index^c^	Not selected (8.1 %)	Not selected (10.8 %)	Not selected (34.2 %)
Rainfall^c^	Not selected (14.4 %)	Not selected (12.3 %)	Not selected (19.6 %)
Rainfall coefficient of variation (CV)	Not selected (7.1 %)	Not selected (17.1 %)	Selected (71.8 %)
Altitude^b,c^	Not selected (23.6 %)	Not selected (42.5 %)	Not selected (39.4 %)
Soil acidity (pH)^b^	Not selected (7.4 %)	Selected (59.0 %)	Not selected (37.2 %)
Soil moisture^a,c^	Not selected (17.7 %)	Selected (90.8 %)	Not selected (22.5 %)
Ecological zone^a,b,c^	Not selected (17.4 %)	Not selected (30.3 %)	Not selected (24.7 %)
Rural/urban setting^a^	Selected (84.3 %)	Not selected (26.2 %)	Not selected (44.6 %)
Human influence index (HII^)b,c^	Not selected (25.3 %)	Not selected (36.1 %)	Not selected (32.7 %)
Improved sanitation	Not selected (23.5 %)	Not selected (23.1 %)	Not selected (19.1 %)
Improved drinking water	Not selected (7.1 %)	Not selected (21.9 %)	Not selected (29.6 %)
Model posterior probability	8.9 %	3.2 %	0.4 %

^a^Categorised for hookworm; ^b^Categorised for *A. lumbricoides*; ^c^Categorised for *T. trichiura*

Model parameter estimates and validation measures of fitted models are provided in Table 4. Parameter estimates show that hookworm infections are favoured in rural settings, as reflected by the negative effect of the variable urban on the log*it* of hookworm risk (odds ratio (OR) = 0.3; 95 % Bayesian credible interval (BCI): 0.2–0.5). Residual spatial correlation was non negligible and estimated to be 217.6 km (95 % BCI: 94.4–585.1 km). Predicted infection risks for *A. lumbricoides* and *T. trichiura* were low throughout Côte d’Ivoire. However, for *A. lumbricoides* infection, we observed a positive effect of soil pH and a positive association of soil moisture on the infection risk. Hence, distribution of *A. lumbricoides* is favoured by moist soils that are too acid with a pH below 5.2, and showed a residual spatial correlation of 75.8 km (95 % BCI: 6.3–429.2 km). We also estimated a positive effect of rainfall CV on *T. trichiura* infection risk (OR = 2.0; 95 %, 95 % BCI: 1.3–3.2), with 20.5 km (95 % BCI: 5.9–80.5 km) as residual spatial correlation estimated. The higher the rainfall variation, the higher the *T. trichiura* infection risk.

**Table 4:** Parameter estimates and validation measures of Bayesian geostatistical models for hookworm, *A. lumbricoides* and *T. trichiura* infection risks in Côte d’Ivoire, late 2011/early 2012

Hookworm	OR (95 % BCI)
Setting	
Rural	1.0
Urban	0.3 (0.2, 0.5)^a^
	Median (95 % BCI)
Variance	1.1 (0.5, 6.3)
Spatial range (km)	217.6 (94.4, 585.1)
	Model validation measures^b^
Mean error (ME) (%)	0.3
Sum standard deviation (SD) (%)	2.2
*A. lumbricoides*	OR (95 % BCI)
Soil acidity (pH)	
< 5.2	1.0
5.2–5.4	0.3 (0.1, 0.9)^a^
≥ 5.4	0.1 (0.0, 0.4)^a^
Soil moisture	2.4 (1.4, 4.0)^a^
	Median (95 % BCI)
Variance	1.5 (0.6, 3.8)
Spatial range (km)	75.8 (6.3, 429.2)
	Model validation measures^b^
Mean error (ME) (%)	−2.1
Sum standard deviation (SD) (%)	1.0
*T. trichiura*	OR (95 % BCI)
Rainfall coefficient of variation (CV)	2.0 (1.3, 3.2)^a^
	Median (95 % BCI)
Variance	2.3 (1.0, 5.0)
Spatial range (km)	20.5 (5.9, 80.5)
	Model validation measures^b^
Mean error (ME) (%)	−0.2
Sum standard deviation (SD) (%)	0.6

^a^Significant based on 95 % BCI

^b^Assessed by fitting the model on a subsample of the data (80 %)

**Fig. 3:**
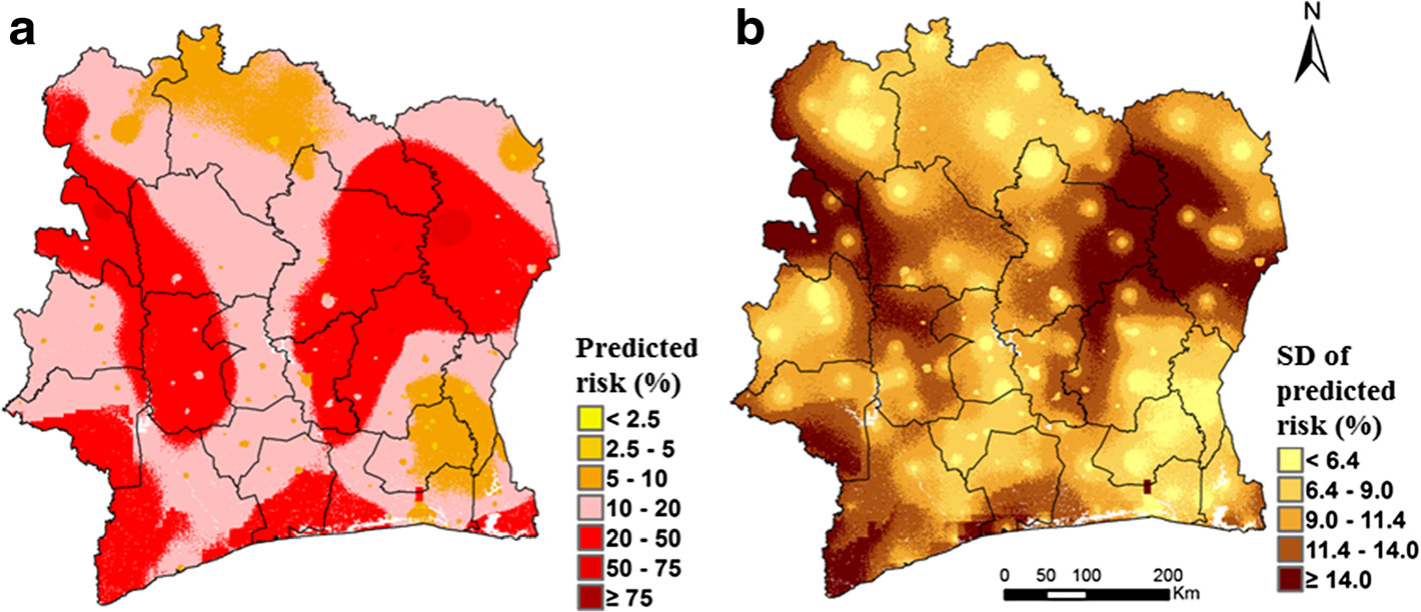
Maps showing the predicted risk (**a**) and standard deviation (SD) of the predictive risk (**b**) of soil-transmitted helminth infections in Côte d’Ivoire, late 2011/early 2012

Figure 3a depicts the overall soil-transmitted helminthiasis predictive risk map for Côte d’Ivoire. Low risk areas (prevalence < 20 %) were mostly concentrated around urban settings. The map of the SD of the model prediction shows that low prediction errors were typically observed around survey locations (Fig. 3b). We estimated an overall soil-transmitted helminth infection children population-adjusted risk of 15.5 % (95 % BCI: 14.2–17.0 %) for Côte d’Ivoire. In particular, the children population-adjusted risks were estimated at 14.1 % (95 % BCI: 12.9–15.4 %) for hookworm infections, 1.5 % (95 % BCI: 1.0–2.5 %) for *A. lumbricoides* infections and 1.1 % (95 % BCI: 0.7–2.1 %) for *T. trichiura* infections, respectively.

**Fig. 4:**
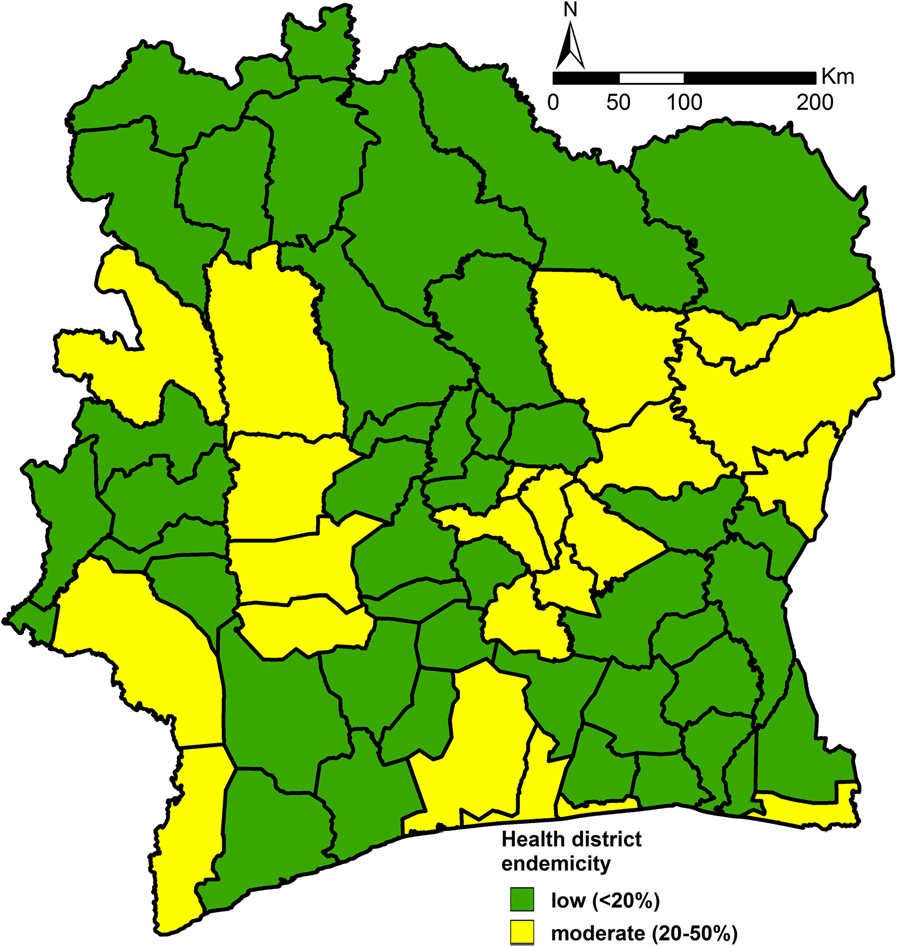
Estimated overall soil-transmitted helminthiasis risk at health district level. Risk is adjusted to the school-aged children population and stratified according to WHO thresholds for intervention planning

Additionally, we provide the school-aged children population-adjusted risk aggregated at health district level as additional information (see Additional file 2). The highest soil-transmitted helminthiasis risk was estimated at 36.1 % for the health district of Nassian, in the north-eastern part of Côte d’Ivoire. A map depicting health districts classified as low (predicted children population-adjusted risk < 20 %) and moderate (predicted children population-adjusted risk between 20 and 50 %) is presented in Fig. 4. According to WHO guidelines that consist to treat once a year all school-aged children living in communities at moderate risk [8], we estimated that a preventive measure implemented at health district level would target 1,290,594 school-aged children. Therefore, the same amount of deworming tablets would be required.

## Discussion

This study presents soil-transmitted helminth infection prevalence data from the first national school-based survey in Côte d’Ivoire and provides model-based estimates of the infection risk at high spatial resolution. Our results show that, in Côte d’Ivoire, hookworm are the predominant soil-transmitted helminths with an estimated school-aged children population-adjusted infection risk of 14.1 %. Only very low prevalence rates were found for *A. lumbricoides* infection (1.5 %) and *T. trichiura* infection (1.1 %). The negative association of urbanisation with hookworm infection risk is consistent with previous studies that highlight that hookworm infections are particularly rampant in rural settings [14, 37].

The prevalence of soil-transmitted helminth infections estimated by our study were lower than expected, especially with regard to *A. lumbricoides* and *T. trichiura* [14]. So far, soil-transmitted helminthiasis risk analyses have been based on historical data at broad scale [37], or with data restricted to well-known endemic areas [11, 38], collected from various sources that employ different methods and techniques [14]. A comparison of issues related to the use of historical data *vs* the use of recent national survey data is provided in the additional information (see Additional files 3 and 4). Thus, our results highlight the importance of a rigorous sampling design, such as the lattice plus close pair design for geostatistical modelling employed in this study [24]. In addition, it should be noted that after the armed conflict ended by the post-electoral crisis [39], the official health programme to control schistosomiasis, soil-transmitted helminthiasis and lymphatic filariasis and non-governmental health organisations restarted and intensified their activities through preventive chemotherapy campaigns targeting school pupils. Furthermore, in the last decades, many places of sub-Saharan Africa are undergoing rapid urbanisation and this development may have a positive impact on the helminth burden thus significantly reducing helminth infection prevalences [40].

We estimated that 1.3 million school-aged children should be dewormed yearly within the frame of preventive chemotherapy programmes implemented at the district level. This corresponds roughly to a third of the number of treatments estimated by Karagiannis-Voules et al. [14]. Indeed, these authors estimated the number of treatments required for school-aged children to amount to 3.6 million. The prior models were based on historical data, which do not necessarily reflect the actual situation, in particular when recent data are sparse [41, 42].

The risk of soil-transmitted helminth infection was estimated below 20 % for most health districts. According to WHO guidelines, school-aged children living in areas with a prevalence lower than 20 %, treatment on case-by-case basis should be undertaken whereas large scale administration of anthelminthic drugs should be advocated in health districts with an estimated moderate risk between 20 and 50 %. Emphasis should also be placed on water, sanitation and hygiene promotion (WASH) to sustain all efforts put in place to control the transmission of soil-transmitted helminthiasis [5, 7, 43].

Our study has several limitations that are offered for consideration. First, we have used the Kato-Katz technique and examined duplicate thick smears from a single stool sample for the presence of eggs of soil-transmitted helminths. Although this technique is recommended by WHO [44], it lacks sensitivity, particularly in low intensity settings. Hence, repeated sampling is required to account for day-to-day variation of egg output [45]. Thus, in our study, light infections were likely missed and the prevalence of soil-transmitted helminths observed may have been underestimated, given that most infections were of light intensity [46]. Secondly, the present study was carried out during the dry season, which could lead to underestimate the prevalence of soil-transmitted helminth infections that occur in Côte d’Ivoire as demonstrated by the effect of rainfall CV on the spread of *T. trichiura*. Thirdly, although the survey covered the entire Côte d’Ivoire, only 92 locations were visited. Increasing the number of schools to be investigated may give a more accurate estimation of soil-transmitted helminth infection prevalence in Côte d’Ivoire.

## Conclusion

To conclude, this study provides smooth maps of the soil-transmitted helminth infection risk among school-aged children in Côte d’Ivoire that reflect well the observed prevalence. In addition, we offer a comprehensive map for the soil-transmitted helminth risk at health district level that can assist health officers controlling the infection according to WHO recommendations. Given that WHO does not recommend large-scale administration of anthelminthic drugs in areas of low soil-transmitted helminth infection, it is essential that monitoring of soil-transmitted helminth infection is regularly done so that treatment strategies can be readily adapted. Finally, although mass drug administration remains the cornerstone to fight against these diseases, we advocate integrated approaches that in addition to preventive chemotherapy aim at improving access to water and sanitation and at changing hygiene behaviour through health education campaigns for the sustainable control and future elimination of these diseases. The latter interventions become even more important when prevalences are very low and individuals with infections need to be treated on a case-by-case basis.

## Additional files

Additional file 1: Standard Bayesian model specification and implementation of variable selectionESM 2 Overall soil-transmitted helminthiasis risk adjusted for school-aged children population (5–15 years old), by health districts. (PDF 87 kb) Additional file 2: Overall soil-transmitted helminthiasis risk adjusted for school-aged children population (5-15 years old), by health districts. (PDF 76 kb) Additional file 3: Map of geographical distribution of data points from the Global Atlas of Helminth Infections (GAHI) database (historical data repository) and the recent national survey in Côte d’Ivoire. (TIF 357 kb) Additional file 4: Advantages and disadvantages of historical data repositories and the recent national cross-sectional survey data in Côte d’Ivoire used for model-based prediction of soil-transmitted helminths. (PDF 82 kb)
