# Supplementary material for: Bayesian risk profiling of soil-transmitted helminth infections and estimates of preventive chemotherapy for school-aged children in Côte d'Ivoire
Source: Parasit Vectors. 2016 Mar 21;9:162. doi: 10.1186/s13071-016-1446-0 (PMC4802658; doi:10.1186/s13071-016-1446-0)
Supplement: Additional file 1: — Standard Bayesian model specification and implementation of variable selectionESM 2 Overall soil-transmitted helminthiasis risk adjusted for school-aged children population (5–15 years old), by health districts. (PDF 87 kb) [file 13071_2016_1446_MOESM1_ESM.pdf]

Additional file 1: Standard Bayesian model specification and implementation of variable selection.

Standard Bayesian geostatistical model

Let's define Y_j as the number of children infected with an helminthiasis (i.e., hookworm, *A. lumbricoides* or *T. trichiura* infection) and n_j as the number of children screened at survey location j, $j = 1,...,92$. We assume that Y_j arise from a Binomial distribution, $Y_j \sim Bin(n_j, p_j)$ and we model the log*it* of the probability of infection p_j as a linear function of covariates X_j and latent spatially structured Gaussian process φ_j, such as: $\log it(p_j) = X_j^T \underline{\beta} + \varphi_j$, where $\underline{\beta}$ is the regression coefficients vector, including a constant. Spatially structured random effects $\underline{\varphi}$ are modelled as a multivariate normal Gaussian effect: $\underline{\varphi} \sim MVN(\underline{0}, \Sigma)$, with variance-covariance matrix Σ, where spatial dependency is introduced *via* an exponential correlation function of Euclidian distances between observed locations, i.e., $\Sigma_{rs} = \sigma^2 \exp(-\rho \; dist_{rs})$, where σ^2 is the spatial variability, ρ control the rate of the spatial decay, and (r, s) is a pair of survey locations. The range is defined as the minimum distance at which spatial correlation between locations is inferior to 5% and is equivalent to $-\log(0.05)/\rho_l$.

Within a Bayesian framework of inference, we considered non-informative prior specification for model parameters. More precisely, we assigned vague normal distribution the regression coefficients $\underline{\beta}$: $\underline{\beta} \sim N(\underline{0}, 100)$, inverse gamma distribution for the spatial variance σ^2: $\sigma^2 \sim IG(2.01, 1.01)$ and gamma distribution for the spatial decay ρ, $\rho \sim G(0.01, 0.01)$.

Stochastic search variable selection

We introduce a binary indicator I_k and a coefficient α_k that express the presence or absence and the size effect of the kth variables in the model ($k = 1,...,16$), respectively. Regression coefficients β_{kl}'s are defined as the product of the indicator I_m and its effect α_{ml}, where $l \in \{1\}$ if the variable k is linear or binary and $l \in \{1,2\}$ if the variable k is categorized into 3 categories. Assuming *a priori* dependence of I_k and α_{kl}, we assigned to I_k a Bernoulli distribution with non-informative Beta distributed hyper-parameter π_k ($I_k \sim Bern(\pi_k)$, $\pi_k \sim Beta(1,1)$). A mixture normal prior distribution is allocated to α_{kl} with a non-informative variance τ_k^2 that follows an inverse-gamma distribution and, if the variable X_k is excluded from the model, is scaled down to shrink the effect size to 0: in case the variable X_k is excluded from the model:

$$\begin{cases} \alpha_{kl} \sim N(0,\tau_k^2), \tau_k^2 \sim IG(5,25) & \textit{if } I_m = 1 \\ \alpha_{kl} \sim N(0,\tau_k^2/4000), \tau_k^2 \sim IG(5,25) & \textit{if } I_m = 0 \end{cases}.$$
