# Supplementary material for: Bayesian risk profiling of soil-transmitted helminth infections and estimates of preventive chemotherapy for school-aged children in Côte d'Ivoire
Source: Parasit Vectors. 2016 Mar 21;9:162. doi: 10.1186/s13071-016-1446-0 (PMC4802658; doi:10.1186/s13071-016-1446-0)
Supplement: Additional file 2: — Overall soil-transmitted helminthiasis risk adjusted for school-aged children population (5-15 years old), by health districts. (PDF 76 kb) [file 13071_2016_1446_MOESM2_ESM.pdf]

Additional file 2: Overall soil-transmitted helminthiasis risk adjusted for school-aged children population (5-15 years old), by health districts.

Health district	Population	Adjusted risk (%)	Health district	Population	Adjusted risk (%)	Health district	Population	Adjusted risk (%)
Nassian	10708	36.1 (29.8; 43.9)	Bouaké-Ouest	17957	19.9 (15.1; 25.1)	Bouaflé	90364	14.8 (10.9; 19.3)
Dabakala	34055	35.9 (29.3; 42.0)	Sakassou	26673	19.6 (15.2; 24.9)	Yamoussoukro	105453	14.7 (9.2; 22.0)
M'Bahiakro	41183	31.2 (25.8; 36.4)	Odienné	33521	19.0 (14.9; 24.7)	Lakota	54978	14.5 (10.2; 19.6)
Touba	37850	31.0 (24.6; 37.7)	Bangolo	44581	18.6 (14.1; 24.1)	Aboisso	112209	13.8 (9.4; 18.9)
Tabou	13161	28.8 (15.0; 51.7)	Beoumi	40392	18.3 (13.7; 23.4)	Gagnoa	149079	13.8 (9.7; 18.7)
Didievi	22217	28.5 (21.1; 36.3)	Bouaké-Est	198225	17.7 (13.2; 24.3)	Dananö	102695	13.5 (8.3; 19.5)
Bondoukou	145400	28.4 (23.9; 33.3)	Zunénoula	54582	17.6 (13.7; 22.8)	Agnibilekro	42481	13.4 (7.8; 21.2)
Grand-Lahou	30082	28.1 (23.3; 33.4)	Dabou	72090	17.5 (13.3; 22.5)	Oumé	69490	13.1 (8.7; 19.6)
Vavoua	102100	27.7 (22.6; 33.8)	Daoukro	53769	17.2 (13.8; 21.3)	Sinfra	63715	12.8 (8.7; 17.5)
Jacqueville	18968	26.8 (20.6; 34.3)	Kounahiri	8932	17.2 (11.0; 23.2)	Bongounanou	114827	12.7 (8.9; 16.6)
Dimbokro	38873	26.1 (20.9; 33.3)	Toulepleu	12941	17.2 (11.9; 23.0)	Grand-Bassam	26643	12.5 (9.1; 17.9)
Adiake	49400	25.9 (20.1; 34.3)	Duekoue	48001	16.9 (10.8; 25.1)	Agboville	104828	12.1 (8.7; 16.2)
Bouaké-Sud	4807	25.8 (19.6; 33.3)	Sassandra	10398	16.9 (11.2; 27.9)	Ferkessédougou	87510	11.7 (8.5; 15.7)
Bocanda	39701	24.8 (20.1; 29.5)	Katiola	47918	16.8 (11.9; 22.2)	Madinani	15332	11.7 (7.7; 16.4)
Tiébissou	25809	24.3 (18.9; 31.3)	Bouna	55176	16.6 (12.4; 21.8)	Boundiali	56277	11.1 (6.2; 16.6)
Koun-Fao	47540	24.0 (18.2; 31.5)	Soubré	58814	16.5 (11.3; 23.6)	Alepe	56547	10.4 (6.1; 15.8)
Daloa	186980	23.4 (19.2; 28.3)	Mankono	50697	16.5 (11.5; 22.6)	Korhogo	163577	10.0 (6.4; 14.7)
Issia	89248	23.4 (18.2; 29.0)	Sikensi	62618	16.5 (12.8; 21.6)	Abengourou	115722	9.3 (6.4; 13.3)
Divo	200712	21.5 (17.6; 26.5)	San-Pedro	44782	16.2 (10.8; 23.2)	Adzopé	75130	8.6 (5.8; 13.2)
Suéguéla	48981	21.1 (16.9; 26.2)	Minignan	19745	15.4 (10.7; 20.2)	Akoupé	46219	7.8 (5.2; 12.2)
Toumodi	42991	20.4 (15.8; 26.6)	Man	120509	15.3 (11.5; 20.5)	Tengréla	21658	7.1 (2.9; 14.8)
Guiglo	59828	20.2 (15.2; 26.6)	Biankouma	39013	15.0 (11.6; 19.3)	Abidjan zone	1048866	6.8 (3.6; 11.2)

Adjusted risks are given with their 95% Bayesian credible intervals (BCI) and are listed in decreasing order of importance.
