# Supplementary figures and images for: Bayesian risk profiling of soil-transmitted helminth infections and estimates of preventive chemotherapy for school-aged children in Côte d'Ivoire
Source: Parasit Vectors. 2016 Mar 21;9:162. doi: 10.1186/s13071-016-1446-0 (PMC4802658; doi:10.1186/s13071-016-1446-0)

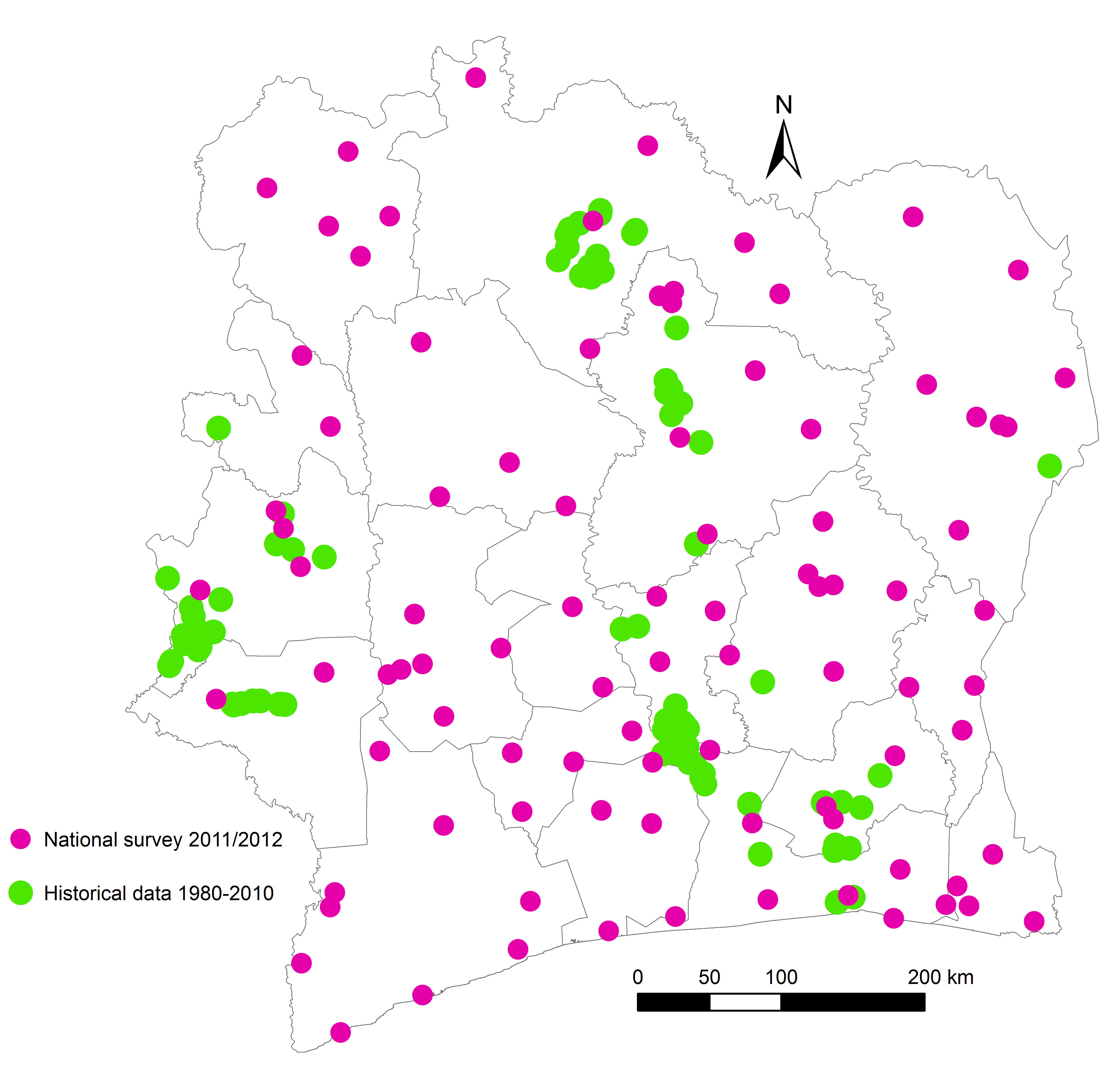

Supplement: Additional file 3: — Map of geographical distribution of data points from the Global Atlas of Helminth Infections (GAHI) database (historical data repository) and the recent national survey in Côte d’Ivoire. (TIF 357 kb) [file 13071_2016_1446_MOESM3_ESM.tif]
