# Supplementary material for: Bayesian risk profiling of soil-transmitted helminth infections and estimates of preventive chemotherapy for school-aged children in Côte d'Ivoire
Source: Parasit Vectors. 2016 Mar 21;9:162. doi: 10.1186/s13071-016-1446-0 (PMC4802658; doi:10.1186/s13071-016-1446-0)
Supplement: Additional file 4: — Advantages and disadvantages of historical data repositories and the recent national cross-sectional survey data in Côte d’Ivoire used for model-based prediction of soil-transmitted helminths. (PDF 82 kb) [file 13071_2016_1446_MOESM4_ESM.pdf]

Table: Advantages and disadvantages of historical data repositories and the recent national cross-sectional survey in Côte d'Ivoire used for model-based prediction of soil-transmitted helminth infections.

Data repository characteristics	Historical data repository		National cross-sectional survey data repository		Publications
	**Description**	**Advantages (+) /disadvantages (-)**	**Description**	**Advantages (+) /disadvantages (-)**	
Sampling design	Clustered, focused on well-known endemic areas	+ Useful in case of data scarcity for model-based prediction for helminth infections.<br>- Overestimation of disease estimates.	Lattice plus close pair design covering the entire country	+ This approach allows to overcome selection bias and to better estimate disease prevalences at country level.	[1–6]
Diagnostics	Different sampling effort and diagnostic approaches, with different objectives and purposes	- Case definition is based on different diagnostic approaches including varying techniques as well as number of examined stool samples per individual. Data are thus not comparable with regard to specificity and sensitivity.	One single approach used: duplicate Kato-Katz thick smears, as defined as standard diagnostic technique recommended by World Health Organization.	+ All data points are comparable in the applied diagnostic approach and the estimated disease prevalence.<br>- Duplicate Kato-Katz thick smears from a single stool sample may not be the most sensitive technique and low-intensity infections thus may be missed.	[3,7,8]
Target population	Age heterogeneity	- Historical data repositories may include data from community-based and school-based surveys. Peak prevalences of helminth infections, however, vary between age groups. Estimates of treatment needs at community-level may be an over- or underestimation depending on the number of individuals in each age strata.	Our study focused on school aged children exclusively, as they are considered as high-risk population for helminth infections.	+ Prevalence rates among the school-aged population serve as indicator for endemicity levels and are used by stakeholders to plan intervention strategies.	[8]

Temporality	Various sources of collection, time heterogeneity and seasonality issue	- Published historical data made available from GAHI platform, are dated back from 1980. This approach is not taking into account the fact that, Côte d'Ivoire underwent a great change, in term of human development and environmental transformation. A national helminth control programme has been set in place meanwhile and deworming and research activities are regularly conducted to mitigate the burden of helminthiasis.	Our database stems from a four-month survey conducted within a single season (i.e. dry season).	+ Helminth distribution is strongly linked to climate predictors. Compared to historical data our data repository represents a cross-sectional cut with regard to respective climatic indicators used for disease estimation and thus allows for a more accurate appraisal of the real situation of soil-transmitted helminth infections in the country during a specific season and allows to overcome potential seasonality effects.	[9–12]

+ Advantages of use of data repository
- Disadvantages of use of data repository

References

[1] Raso G, Vounatsou P, Gosoniu L, Tanner M, N'Goran EK, Utzinger J. Risk factors and spatial patterns of hookworm infection among schoolchildren in a rural area of western Côte d'Ivoire. Int J Parasitol. 2006; 36: 201–210.

[2] Sturrock HJW, Gething PW, Ashton RA, Klaczinski JH, Kabatereine NB, Brooker SJ. Planning schistosomiasis control: investigation of alternative sampling strategies for *Schistosoma mansoni* to target mass drug administration of praziquantel in East Africa. Int Health. 2011; 3: 165–175.

[3] Pullan RL, Sturrock HJW, Magalhães RJS, Clements ACA, Brooker SJ. Spatial parasite ecology and epidemiology: a review of methods and applications. Parasitology. 2012; 139: 1870–1887.

[4] J. Coulibaly JT, Fürst T, Silué KD, Knopp S, Hauri D, Ouattara M, Utzinger J, N'Goran EK. Intestinal parasitic infections in schoolchildren in different settings of Côte d'Ivoire: effect of diagnostic approach and implications for control. Parasit Vectors. 2012; 5: 135.

[5] A. Righetti AA, Glinz D, Adiossan LG, Koua AYG, Niamké S, Hurrell RF et al. Interactions and potential implications of *Plasmodium falciparum*-hookworm coinfection in different age groups in South-Central Côte d'Ivoire. PLoS Negl Trop Dis. 2012; 6: e1889.

[6] Karagiannis-Voules DA, Biedermann P, Ekpo UF, Garba A, Langer E, Mathieu E et al. Spatial and temporal distribution of soil-transmitted helminth infection in sub-Saharan Africa: a systematic review and geostatistical meta-analysis. Lancet Infect Dis. 2015; 15: 74–84.

[7] Utzinger J, Booth M, N'Goran EK, Müller I, Tanner M, Lengeler C. Relative contribution of day-to-day and intra-specimen variation in faecal egg counts of *Schistosoma mansoni* before and after treatment with praziquantel. Parasitology. 2001; 122: 537–544.

[8] WHO. Helminth control in school-age children: A guide for managers of control programmes, second edition. Geneva: World Health Organization. 2011.

[9] Hotez PJ. Hookworm and poverty. Ann N Y Acad Sci. 2008; 1136: 38–44.

[10] Tchuenté LAT, N'Goran EK. Schistosomiasis and soil-transmitted helminthiasis control in Cameroon and Côte d'Ivoire: implementing control on a limited budget. Parasitology. 2009; 136: 1739–1745.

[11] F. Chammartin, Guimarães LH, Scholte RGC, Bavia ME, Utzinger J, Vounatsou P. Spatio-temporal distribution of soil-transmitted helminth infections in Brazil. Parasit Vectors. 2014; 7: 440.

[12] Riess H, Clowes P, Kroidl I, Kowuor DO, Nsojo A, Mangu C et al. Hookworm Infection and environmental factors in Mbeya region, Tanzania: a cross-sectional, population-based study. PLoS Negl Trop Dis. 2013; 7: e2408.
